# Unraveling the gut microbiota’s role in PCOS: a new frontier in metabolic health

**DOI:** 10.3389/fendo.2025.1529703

**Published:** 2025-03-18

**Authors:** Caihong Li, Dongkai Cheng, Haiqin Ren, Tao Zhang

**Affiliations:** ^1^ Department of Assisted Reproductive Laboratory, Shenyang Jinghua Hospital, Shenyang, China; ^2^ Department of Stem Cells and Regenerative Medicine, Shenyang Key Laboratory of Stem Cell and Regenerative Medicine, China Medical University, Shenyang, China

**Keywords:** polycystic ovary syndrome, gut microbiota, insulin resistance, metabolic disorders, fecal microbiota transplantation

## Abstract

Polycystic ovary syndrome (PCOS) is a common endocrine and metabolic disorder affecting reproductive-age women, characterized primarily by hyperandrogenism, ovulatory dysfunction, and metabolic abnormalities. In recent years, the gut microbiota has garnered widespread attention for its potential role as a key regulator of host metabolism in the pathogenesis of PCOS. Studies have shown that PCOS patients exhibit dysbiosis in their gut microbiota, characterized by reduced microbial diversity, an imbalance in the ratio of Firmicutes to Bacteroidetes, changes in the abundance of specific taxa, and abnormal levels of metabolic products. These alterations may exacerbate metabolic dysfunction in PCOS through multiple mechanisms, including influencing host energy metabolism, disrupting lipid and bile acid metabolism, and inducing chronic inflammation. Addressing gut dysbiosis through the modulation of patients’ microbiomes—such the use of, prebiotics, fecal microbiota transplantation, and optimizing diet lifestyle—may offer strategies for improving metabolic abnormalities and alleviating clinical symptoms in PCOS. Additionally, the gut microbiome promises as a potential marker, aiding in the precise diagnosis and personalization of PCOS. Although our current understanding of how the gut microbiota influences PCOS is still limited, research is needed to explore the causal relationships and mechanisms involved, providing a more reliable theoretical basis for clinical. This review aims summarize the research progress on the relationship between gut microbiota and PCOS, and to suggest future directions to promote the development of prevention and treatment strategies for PCOS.

## 1 Introduction

Polycystic ovary syndrome (PCOS) is a prevalent endocrine and metabolic disorder affecting women of reproductive age, characterized by hyperandrogenism, ovulatory dysfunction, and various metabolic abnormalities. These features can significantly impact the reproductive health and quality of life of affected individuals. Epidemiological studies suggest that approximately 5-10% of women of reproductive age globally are affected by PCOS (1). The exact pathogenesis of PCOS remains incompletely understood; however, it is widely believed to arise from a combination of genetic, environmental, and lifestyle factors (2). Genetic influences are evident in the familial clustering of PCOS cases, indicating a potential polygenic basis for the disorder; however, isolated single-gene mutations are insufficient to account for the condition. Environmental factors also play a crucial role, with unhealthy dietary habits, physical inactivity, obesity, and excessive hormone exposure during fetal development identified as possible triggers or exacerbating factors (3). In the context of PCOS, hyperandrogenism and insulin resistance are recognized as central features. Hyperandrogenism refers to the abnormal elevation of serum androgen levels (such as testosterone and androstenedione), which can lead to ovulatory disorders, impairing follicular development in the ovaries and resulting in the characteristic “polycystic” appearance (4). Moreover, insulin resistance is observed in many patients with PCOS, influencing glucose metabolism and further stimulating the ovaries to produce androgens, which exacerbates hyperandrogenic symptoms (5). Therefore, it is clear that PCOS emerges from a complex interplay of multiple mechanisms, including endocrine dysfunction and abnormalities in insulin metabolism, which collectively have profound effects on women’s reproductive health and metabolic function.

Obesity is a common complication of PCOS, and gut microbiota dysbiosis is closely related to the development of obesity. It exacerbates obesity and its associated metabolic disorders by affecting fat storage and energy metabolism (6). Genetic factors and metabolic defects play a crucial role in the onset and progression of obesity and further exacerbate obesity and its related metabolic disturbances by influencing the gut microbiota (7). Studies have shown that several obesity-related genes (such as appetite regulation genes: FTO, MC4R, LEP, LEPR; energy metabolism genes: ADRB3, PPARG, UCP1; insulin resistance genes: TCF7L2, IRS1; biological clock and neuroregulatory genes: CLOCK, BDNF) have mutations or polymorphisms that affect appetite regulation, fat storage, energy expenditure, and metabolic function, increasing the individual’s susceptibility to obesity (8–13). However, genetic factors are not the only mechanism leading to obesity.

Metabolic abnormalities also play a critical role in obesity. For example, insulin resistance, metabolic syndrome, and lipid metabolism disorders are often closely associated with obesity, especially in PCOS patients, where hyperinsulinemia further promotes fat storage and weight gain (14). In addition, metabolic abnormalities may affect adipocyte function and fat tissue distribution, leading to excessive visceral fat accumulation, which aggravates obesity. Obesity is also closely related to gut microbiota dysbiosis. Studies have found that obesity significantly alters the composition and function of the gut microbiota, and this microbial imbalance may exacerbate metabolic disorders, creating a vicious cycle (15). For example, gut microbiota dysbiosis may impair gut barrier function, leading to leaky gut, which triggers systemic inflammation and decreases insulin sensitivity (16).

Furthermore, changes in the gut microbiota not only affect metabolic regulation but also induce a series of pathological processes through other mechanisms, such as immune dysfunction, leading to chronic low-grade inflammation. This inflammation is prevalent in obesity, PCOS, and other metabolic diseases and further exacerbate insulin resistance (17). Notably, the gut microbiota can also influence the gut-brain axis through its metabolites (such as short-chain fatty acids), thereby regulating appetite and mood, which is particularly evident in obese individuals. There exists a complex interaction between genetic defects, metabolic abnormalities, and the gut microbiota. Metabolic disorders alter the composition of the gut microbiota, while gut microbiota dysbiosis may worsen metabolic disturbances, promoting the progression of obesity. Therefore, exploring the role of gut microbiota in obesity caused by genetic and metabolic abnormalities not only helps understand the mechanisms behind obesity but also provides new research directions and potential therapeutic targets for obesity and related metabolic diseases.

The microbiome is the group of microbes (like bacteria, viruses, and fungi) living in and on the body. These microbes help with things like digestion, protecting against harmful germs, and supporting the immune system (18). In recent years, advancements in technologies like metagenomic sequencing have significantly enhanced our understanding of the role of gut microbiota in human health and disease (19, 20). Research has established that gut microbiota plays a crucial role in regulating various physiological processes in the host, including nutrient metabolism, immune responses, and neuroendocrine functions. An imbalance in gut microbiota has been closely linked to several chronic diseases, such as obesity, type 2 diabetes, and cardiovascular diseases (21). Given the notable metabolic abnormalities associated with PCOS, an increasing number of studies have begun to explore the potential role of gut microbiota in the pathogenesis of this condition (22). A substantial body of evidence suggests that PCOS patients often exhibit gut microbiota dysbiosis, characterized primarily by reduced microbial diversity, imbalances in dominant microbial populations, and changes in metabolic byproducts (23–25). Gut dysbiosis may influence the progression of PCOS through various mechanisms. For instance, an imbalance in gut microbiota could alter the host’s metabolic state, contributing to metabolic abnormalities in PCOS by inducing low-grade chronic inflammation (26). Xu et al. found significant differences in the gut microbiota structure of PCOS patients compared to healthy individuals, particularly noting that reduced levels of certain beneficial bacteria may compromise gut barrier function, thereby heightening the risk of metabolic disturbances (27). Additionally, levels of gut microbiota metabolic byproducts, such as short-chain fatty acids, are found to be lower in PCOS patients, which can adversely affect insulin sensitivity and further exacerbate the metabolic issues associated with PCOS (28).

A deeper exploration of the relationship between gut microbiota and PCOS may enhance our understanding of the disorder’s pathogenesis from a microecological perspective, offering new insights for its prevention and treatment. This article aims to systematically summarize recent research advancements concerning the relationship between gut microbiota and PCOS. We will focus on the characteristic alterations in the gut microbiota of PCOS patients, the potential mechanisms through which gut dysbiosis may influence metabolic abnormalities associated with PCOS, and the prospects for regulating gut microbiota as a strategy for managing the condition. Additionally, we will underscore future research directions to provide reference and inspiration for elucidating the role of gut microbiota in PCOS.

## 2 Characteristics of gut microbiome changes in PCOS patients

Current studies indicate significant differences in both the composition and metabolic activity of the gut microbiome between individuals with PCOS and healthy women. These differences are primarily characterized by the following aspects:

### 2.1 Reduced diversity

Numerous studies have reported a markedly lower microbial alpha diversity index in fecal samples from PCOS patients compared to healthy controls (24, 29–31). For instance, Torres et al. (32) conducted an analysis involving 73 PCOS patients and 48 healthy controls using 16S rRNA gene sequencing, revealing that the alpha diversity of the gut microbiome in women with PCOS was significantly decreased. This reduction in alpha diversity was also found to correlate negatively with hyperandrogenemia, total testosterone levels, and hirsutism. Similarly, a study by Insenser et al. (33) reported analogous findings involving 15 PCOS patients and 16 healthy controls. Additionally, women with PCOS demonstrated specific alterations in their gut microbiome, including an increased abundance of Actinobacteria and Candidatus. The observed reduction in microbial diversity suggests a decline in the stability and resilience of the gut microecology in PCOS patients, rendering them more vulnerable to external factors and exacerbating existing imbalances.

### 2.2 Increased ratio of bacteroidetes to firmicutes

Bacteroidetes and Firmicutes are the two predominant phyla of gut microbiota in the human intestine, and an imbalance in their ratio has been linked to various metabolic diseases (34, 35). In a study by Lindheim et al. (36), fecal samples from 24 PCOS patients (consisting of 12 obese and 12 non-obese individuals) and 19 healthy controls were analyzed. The researchers found that the abundance of Bacteroidetes was significantly higher in the PCOS group, while the abundance of Firmicutes was notably lower, resulting in an increased Bacteroidetes-to-Firmicutes ratio. Similar findings were reported in Liu et al.’s study (37), which involved 33 PCOS patients and 15 healthy controls. The excessive proliferation of Bacteroidetes indicates an imbalance in the gut microbiota among PCOS patients, potentially leading to pathogenic implications by negatively affecting host metabolism and immune responses.

### 2.3 Altered abundance of specific bacteria

Significant changes have also been observed in the abundance of certain bacterial genera within the guts of PCOS patients (38, 39). In a study conducted by Qi et al. (20), metagenomic sequencing of fecal samples from 50 PCOS patients and 43 healthy controls revealed a marked reduction in beneficial bacteria such as Akkermansia and Bifidobacterium in the PCOS group. Conversely, there was a notable increase in the abundance of Desulfovibrio, a genus associated with metabolic abnormalities and inflammation. Additionally, Insenser et al. (33) reported a decrease in the abundance of Prevotella among PCOS patients. These alterations signify a decline in beneficial gut flora and an increase in potentially harmful bacteria, contributing to the overall microbial imbalance in PCOS.

### 2.4 Changes in firmicutes composition

The Firmicutes phylum comprises several genera, including Lactobacillus, Clostridium, and Ruminococcus (40). In addition to the overall reduction in Firmicutes abundance, variations in the internal composition of this phylum have also been observed in PCOS patients. In their analysis of the gut microbiome structure, Liu et al. (37) noted an increase in the abundance of Ruminococcus and Coprococcus within the Firmicutes phylum among PCOS patients. This finding sharply contrasts with the composition observed in healthy controls, suggesting that the dysregulation of specific bacterial populations in PCOS may be linked to particular metabolic abnormalities. The study indicates that the increased relative abundance of Ruminococcus and Coprococcus within Firmicutes could play a significant role in the pathological development of PCOS by influencing the host’s short-chain fatty acid metabolism and pro-inflammatory signaling pathways. Similarly, Zeng et al. (41) reported a significant increase in the abundance of both Ruminococcus and Coprococcus in the guts of PCOS patients. This proliferation of specific bacterial genera highlights a disruption in the internal balance of Firmicutes, reflecting a distinct pattern of metabolic imbalance within the microbiome structure of PCOS. The increased abundance of these genera may further impact the overall metabolic functions of the gut microbiota.

### 2.5 Changes in metabolite levels

The gut microbiome produces a variety of bioactive substances through its metabolic activities, including short-chain fatty acids, amines, and phenolic compounds, which play essential roles in regulating host physiological functions (42). Evidence of metabolic dysregulation within the gut microbiome of PCOS patients is steadily accumulating (43). For instance, Zeng et al. (41) found that levels of short-chain fatty acids, such as butyrate, were significantly lower in the feces of PCOS patients compared to healthy controls. Additionally, Qi et al. (23) conducted metagenomic and metabolomic analyses, revealing a significant enrichment of genes related to amino acid metabolism in the gut microbiota of PCOS patients, along with elevated plasma levels of branched-chain amino acids (BCAAs). It is important to note that PCOS patients often present with obesity, hyperglycemia, and dyslipidemia. Some studies have reported a positive correlation between plasma BCAA levels and markers of metabolic syndrome, such as weight, body mass index (BMI), and insulin sensitivity (44, 45). These abnormal changes in metabolite levels reflect dysfunction within the gut microbiome of PCOS patients and play a crucial role in the progression of the disease.

Overall, existing studies strongly support the idea that dysbiosis of the gut microbiome is present in PCOS patients, characterized by reduced microbial diversity, an increased Bacteroidetes-to-Firmicutes ratio, altered abundance of specific bacteria, changes in Firmicutes composition, and abnormal metabolite levels (see **Table 1**). These alterations may contribute to the pathogenesis of PCOS by affecting the host’s metabolism, inflammation, and other biological pathways. However, it is important to note that there is some heterogeneity across different studies, and the causal relationship between gut microbiome dysbiosis and the development of PCOS warrants further investigation.

**Table 1:** Summarizes the main changes in the gut microbiota of PCOS patients at the phylum, genus, and metabolite levels, as well as the mechanisms through which these changes may affect PCOS.

Microbiota Changes	Mechanism of Influence	References
Phylum Level		
Increased Bacteroidetes abundance	Associated with metabolic disorders such as obesity and insulin resistance	Lindheim et al. (36), Liu et al. (37)
Decreased Firmicutes abundance	Associated with metabolic disorders such as obesity and insulin resistance	Lindheim et al. (36), Liu et al. (37)
Increased Bacteroidetes/Firmicutes ratio	Associated with metabolic disorders such as obesity and insulin resistance	Lindheim et al. (36), Liu et al. (37)
Genus Level		
Decreased Akkermansia abundance	Weakens gut barrier function, promotes inflammation	Qi et al. (23)
Decreased Bifidobacterium abundance	Weakens gut barrier function, promotes inflammation	Qi et al. (23)
Increased Desulfovibrio abundance	Produces hydrogen sulfide, inducing inflammation and oxidative stress	Qi et al. (23)
Decreased Prevotella abundance	Associated with metabolic abnormalities in PCOS	Insenser et al. (33)
Increased Ruminococcus abundance	Influences energy metabolism and gut barrier function	Liu et al. (37)
Increased Coprococcus abundance	Influences energy metabolism and gut barrier function	Liu et al. (37)
Metabolite Level		
Decreased short-chain fatty acid (e.g., butyrate) production	Affects energy metabolism, gut barrier function, and regulation of the hypothalamic-pituitary-ovarian axis	Zeng et al. (41), He et al. (30)
Increased levels of amino acid metabolites (e.g., branched-chain amino acids)	Associated with metabolic abnormalities in PCOS	Qi et al. (23)

These mechanisms primarily include the induction of metabolic disorders, promotion of inflammatory responses, impairment of gut barrier function, and interference with neuroendocrine regulation.

## 3 Mechanisms by which gut microbiota dysbiosis promotes metabolic abnormalities in PCOS

**Figure 1:**
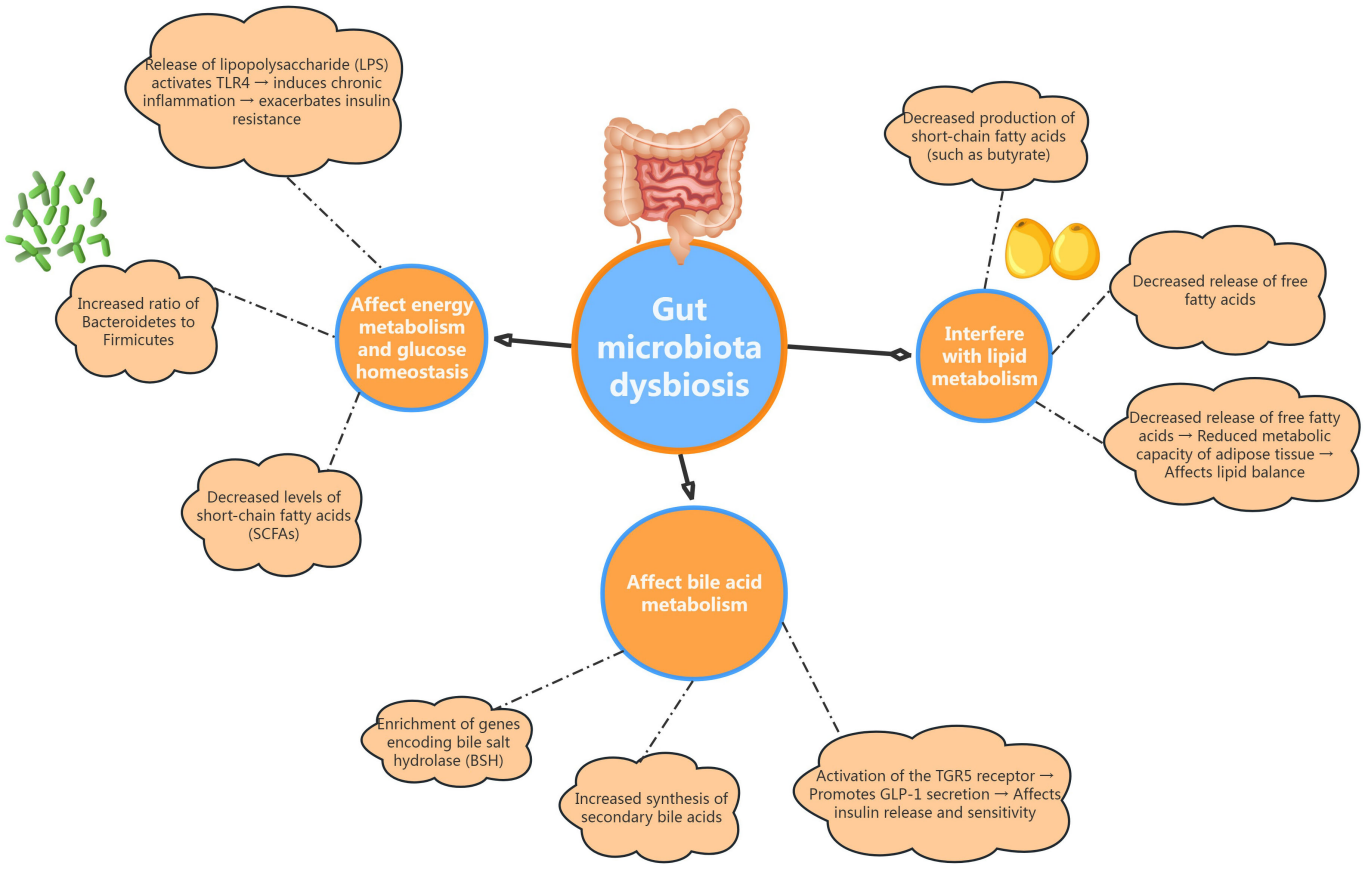
Illustrates how gut dysbiosis impacts energy metabolism and glucose homeostasis, interfere with lipid metabolism, and affects bile acid metabolism in PCOS patients.

Gut microbiota dysbiosis may influence the metabolic homeostasis of patients with PCOS through various mechanisms, potentially promoting the onset of metabolic conditions such as insulin resistance, glucose metabolic disorders, and disturbances in lipid metabolism (see **Figure 1**).

### 3.1 Influence on energy metabolism and glucose homeostasis

Patients with PCOS exhibit distinct alterations in their gut microbiota, notably characterized by an increased Bacteroidetes-to-Firmicutes ratio, which is closely linked to obesity and insulin resistance (46). Within the Bacteroidetes phylum, certain genera, such as Prevotella, demonstrate strong carbohydrate fermentation capabilities, enhancing the production of short-chain fatty acids (SCFAs) that play a vital role in regulating the host’s energy balance (47). However, SCFA levels in the gut of PCOS patients are often reduced, which may diminish the beneficial effects these compounds have on host metabolism. Moreover, in rodent studies, gut microbiota dysbiosis can activate pattern recognition receptors, such as Toll-like receptor 4 (TLR4), through the release of lipopolysaccharides (LPS) and other bacterial toxins. This activation can initiate a chronic inflammatory response, disrupt insulin signaling pathways, and exacerbate insulin resistance in peripheral tissues (48, 49). Overall, dysbiosis of the gut microbiota significantly contributes to glucose metabolic abnormalities in PCOS patients by adversely influencing host energy metabolism and promoting chronic inflammation.

### 3.2 Interference with lipid metabolism

Changes in gut microbiota among PCOS patients significantly influence lipid metabolism through various mechanisms (50). On one hand, alterations in the composition of Firmicutes can lead to a decreased production of SCFAs, such as butyrate. SCFAs are not only a primary energy source for intestinal epithelial cells, but also play a crucial role in inhibiting lipolysis in adipose tissue by activating G protein-coupled receptor GPR43. This activation helps reduce the release of free fatty acids, thereby improving insulin sensitivity in peripheral tissues and maintaining lipid homeostasis (51). Therefore, the reduced levels of SCFAs may be a significant factor contributing to lipid metabolic abnormalities in PCOS patients. On the other hand, the decreased abundance of bacteria associated with lipid synthesis, such as Prevotella, may impair the gut’s ability to process and metabolize dietary fats. This disruption can further negatively impact the host’s lipid balance, exacerbating lipid metabolic dysregulation in individuals with PCOS (52).

### 3.3 Impact on bile acid metabolism

Bile acids, which are steroid compounds derived from cholesterol, play a critical role in the digestion and absorption of lipids (53, 54). Recent studies have also revealed that bile acids have endocrine functions, regulating glucose and lipid metabolism. The gut microbiota is responsible for converting primary bile acids into secondary bile acids through the action of various enzymes, including bile salt hydrolases (BSH). Secondary bile acids exhibit increased hydrophobicity and signaling activity compared to their primary counterparts (55, 56). Research by Qi et al. (23) has demonstrated that genes encoding BSH are significantly enriched in the gut microbiome of PCOS patients, leading to elevated synthesis of secondary bile acids. This increase may promote the secretion of GLP-1 and other incretins from intestinal L-cells by activating TGR5 receptors, which in turn influences insulin secretion and enhances insulin sensitivity in peripheral tissues. Therefore, dysregulated bile acid metabolism mediated by gut microbiota may represent another crucial factor contributing to the metabolic abnormalities with PCOS.

## 4 Application prospects of modulating gut microbiota in the treatment of PCOS

Given the significant role of gut microbiota dysbiosis in the pathogenesis of PCOS, targeted modulation of the gut microecology in affected patients holds promise as a novel treatment strategy. This approach aims to improve metabolic abnormalities and alleviate clinical symptoms, demonstrating broad potential for application in clinical practice.

### 4.1 Gut microbiota as a novel biomarker for disease subtyping and treatment prediction

PCOS is characterized by substantial heterogeneity, with marked differences in clinical phenotypes, metabolic characteristics, and treatment responses among patients (57). The gut microbiome, a key regulator of host metabolic activities, presents opportunities for its use as a novel biomarker for assessing PCOS subtypes and predicting treatment outcomes (58, 59). For example, Liu et al. (36) conducted metagenomic sequencing of the gut microbiota in PCOS patients and healthy controls, developing a diagnostic model based on 61 genera that effectively distinguishes PCOS from healthy individuals. Integrating the characteristics of patients’ gut microbiomes could facilitate precise diagnosis and stratified treatment approaches for PCOS, leading to more personalized treatment plans. In the future, it may be beneficial to explore the incorporation of fecal microbiota sequencing and other advanced technologies into routine auxiliary examinations for PCOS, which could guide clinical decision-making and enhance treatment efficacy.

### 4.2 The application of probiotics and prebiotics as dietary supplements

Probiotics and prebiotics are emerging as potential “biological agents” for improving gut microecology and addressing metabolic abnormalities in PCOS patients (60, 61). Traditional probiotic formulations typically include common strains such as lactobacilli and bifidobacteria. While these probiotics can partially alleviate clinical symptoms and enhance metabolic indicators in PCOS patients, their overall efficacy tends to be limited (62). One study found that prebiotic intake increases the abundance of bifidobacteria in the colon and enhances the production of GLP-1 by colonic L cells, thereby helping to improve insulin resistance (63). Certain gut microbes can synthesize and secrete gamma-aminobutyric acid (GABA) (64). For instance, some strains of lactobacilli and bifidobacteria have been shown to produce GABA, providing a theoretical basis for the role of gut microbiota in modulating GABA levels (65–67). Given that many PCOS patients experience insulin resistance and metabolic syndrome, research suggests that GABA may play a role in regulating energy metabolism, potentially influencing insulin secretion and its utilization, thus impacting metabolic health in this population (68, 69).

Considering the specific changes in gut microbiota associated with PCOS, future research may identify more targeted beneficial strains for developing new probiotic formulations. For example, Qi et al. (20) demonstrated that transferring fecal microbiota from healthy individuals to PCOS mice significantly improved their metabolic disorders and ovarian function. This finding suggests that supplementing with key beneficial strains that are deficient in PCOS patients represents a promising approach for developing effective probiotic interventions. Additionally, the combined administration of probiotics with prebiotics and dietary fibers is expected to further enhance these regulatory effects (44).

### 4.3 Exploratory clinical applications of fecal microbiota transplantation

FMT is a therapeutic approach that aims to restore gut microecology by transferring fecal microbiota from healthy donors to patients (70–73). The gut microbiota plays a crucial role in maintaining host immunity, metabolic balance, and preventing disease. Dysbiosis of the gut microbiota has been linked to various conditions, including obesity, diabetes, and cardiovascular diseases (74–76). Recent studies have revealed a significant association between gut microbiota dysbiosis and the pathogenesis of PCOS, positioning FMT as a promising strategy for restoring microbiota balance in affected individuals (77).

In animal models of PCOS, the administration of fecal microbiota from healthy control mice has demonstrated a notable reduction in alpha diversity and a shift in beta diversity toward levels observed in the control group (78). These findings align with prior studies indicating that FMT using microbiota from healthy individuals can restore a healthy gut microbiome composition in letrozole-induced PCOS mice. FMT has been shown to effectively alleviate metabolic disorders and polycystic ovarian changes in PCOS rats (79, 80).

Zhang et al. conducted a comparison of the effects of short-term FMT (administered three times a week for two weeks) with traditional treatments such as probiotics, contraceptives, and berberine on gut microbiota and metabolic phenotypes in PCOS rats (81). The results indicated that short-term FMT could partially improve metabolic abnormalities and regulate gut microbiota diversity, although its efficacy was less pronounced compared to long-term probiotic supplementation. This suggests that FMT for treating PCOS may require a longer duration or need to be combined with other therapeutic interventions for optimal effectiveness.

Currently, research on FMT for PCOS is largely at the animal experimental stage, providing preliminary evidence for the feasibility of FMT as a method to reshape gut microbiota and regulate metabolic and endocrine disorders. However, further human clinical trials are essential to validate the specific efficacy, dosing regimens, and underlying mechanisms of action (82, 83). Future studies should aim to explore the optimal protocols for FMT treatment in PCOS, evaluate long-term benefits and potential risks, and refine clinical application processes to offer new therapeutic options for PCOS patients. As microbiome and metabolomics technologies continue to advance, the role of FMT in the treatment of PCOS is expected to be more effectively understood and utilized in clinical practice.

Although FMT is considered a relatively safe treatment method, it still carries certain risks. Firstly, even with strict donor screening, FMT still introduces unknown pathogens, such as bacteria, viruses, fungi, or parasites (84). Secondly, the recipient has an immune response to the donor microbiota, leading to chronic inflammation or exacerbation of pre-existing autoimmune diseases (85). Furthermore, FMT triggers abnormal immune regulation, affecting the low-grade inflammation state in PCOS patients (86). In addition, studies have found that transplanting fecal microbiota from obese donors may lead to weight gain in the recipient, suggesting that FMT can affect metabolic balance (87).

### 4.4 Comprehensive application of dietary and lifestyle interventions

A healthy diet and lifestyle are crucial for maintaining gut microecological balance (88, 89). BCAAs, which are major constituents of protein, can aid in weight management (90). Comprising valine, leucine, and isoleucine, BCAAs possess metabolic properties that promote protein synthesis. Research by Lotta et al. (91) has indicated that a higher intake of branched-chain amino acids is associated with an increased risk of developing type 2 diabetes (92). Notably, fluctuations in valine and leucine levels are closely related to insulin resistance in both lean and obese PCOS patients.

**Figure 2:**
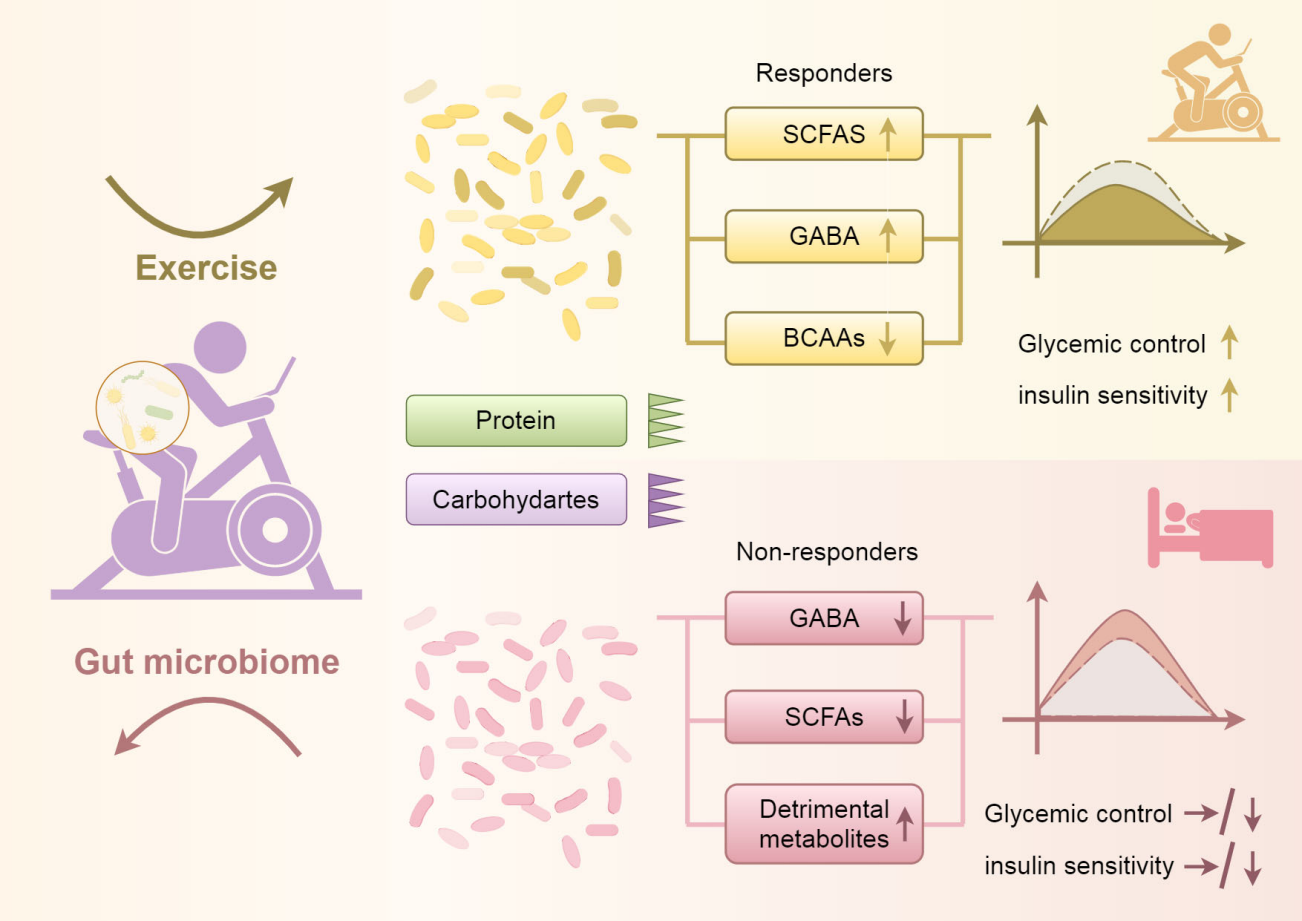
Shows that increasing aerobic exercise and reducing protein and carbohydrate intake can lead to changes in the gut microbiota, resulting in decreased levels of BCAAs, increased levels of SCFAs, and elevated levels of GABA. These changes contribute to improved glycemic control and enhanced insulin sensitivity. Conversely, reducing aerobic exercise and increasing protein and carbohydrate intake can cause alterations in the gut microbiota, leading to decreased levels of SCFAs and GABA, and possibly resulting in an increase in harmful metabolic byproducts, which in turn can lead to poorer glycemic control and reduced insulin sensitivity.

Studies have shown that increased consumption of animal protein can stimulate the growth of certain bacteria, such as Bacteroides, Alistipes, and bifidobacteria, while decreasing the abundance of Bifidobacteria in adolescents. Conversely, an increase in plant protein intake has been linked to a promotion of beneficial bacteria such as Bifidobacterium and Lactobacillus, a reduction in the fragile populations of Bacteroides and Clostridium perfringens, and an enhancement in the production of SCFAs (77, 93). Reducing animal protein intake while increasing aerobic exercise can lead to lowered levels of BCAAs and elevated levels of SCFAs. This shift is expected to improve glycemic control and enhance insulin sensitivity among individuals with PCOS (see **Figure 2**). Integrating these dietary and lifestyle interventions can support overall metabolic health and contribute to better management of PCOS symptoms.

Therefore, integrating personalized dietary guidance and exercise prescriptions with traditional lifestyle interventions and innovative strategies such as probiotics is anticipated to optimize gut microbiota in PCOS patients and enhance their metabolic and endocrine homeostasis. This approach necessitates collaboration among multidisciplinary experts in nutrition, exercise medicine, microbiology, and other fields to develop a comprehensive management plan tailored for individuals with PCOS (94–96). For instance, dietary recommendations could prioritize high-fiber, low-saturated fat diets, while exercise prescriptions might focus on moderate-intensity aerobic activities, complemented by the use of probiotics. This synergistic combination is expected to collectively improve both the gut microbiota and clinical outcomes for patients (97, 98).

### 4.5 Potential application value of vagus nerve stimulation

The vagus nerve plays an important role in regulating the gut microbiota and treating PCOS, mainly through the gut-brain axis to influence insulin sensitivity, inflammation levels, hormonal balance, and neural function (99). PCOS patients often exhibit gut microbiota dysbiosis, characterized by an increase in harmful bacteria and a decrease in beneficial bacteria, which may lead to compromised gut barrier function (leaky gut), exacerbated systemic inflammation, and insulin resistance, thereby aggravating the metabolic disturbances and hyperandrogenemia in PCOS.

The vagus nerve regulates inflammation levels through the cholinergic anti-inflammatory pathway (CAP), where acetylcholine (ACh) released by the vagus nerve can suppress pro-inflammatory cytokines (TNF-α, IL-6), reduce chronic low-grade inflammation, and help improve insulin sensitivity, decrease fat storage, and enhance energy metabolism (100). Additionally, the vagus nerve can promote the secretion of hormones such as GLP-1 (glucagon-like peptide-1) and PYY (peptide YY), which improves insulin resistance, helps regulate blood glucose and body weight in PCOS patients. Furthermore, the vagus nerve can influence the HPO axis (hypothalamic-pituitary-ovarian axis), regulate estrogen and androgen levels, and promote the restoration of ovulatory function, thereby alleviating menstrual irregularities and infertility in PCOS (101). In terms of neural regulation, the vagus nerve also influences mood by modulating serotonin (5-HT) and GABA, improving common anxiety and depression symptoms in PCOS patients (102). Clinical studies have found that vagus nerve dysfunction may exacerbate mood disorders related to PCOS, while VNS can enhance vagus nerve activity, improve neurotransmitter regulation, and thus alleviate mood disorders, further affecting metabolism and hormone levels through the “gut-brain-hormone axis” (103).

VNS is an emerging neuroregulatory treatment that, by modulating gut microbiota, improving insulin resistance, reducing inflammation, and regulating hormone levels, may provide a new therapeutic approach for PCOS and its associated metabolic abnormalities (104). Although VNS is still in the research stage in the field of PCOS, it shows potential value in improving endocrine function, inflammation, and mood disorders.

## 5 Summary and outlook

In summary, extensive research has demonstrated that patients with PCOS frequently experience gut dysbiosis, characterized primarily by reduced microbial diversity, imbalances in dominant microbial populations, and disrupted metabolic activity. These alterations may significantly contribute to the onset and progression of PCOS by affecting host energy metabolism, inducing chronic inflammation, and interfering with lipid and bile acid metabolism. Targeted interventions aimed at regulating gut microecology, such as the supplementation of probiotics and prebiotics, fecal microbiota transplantation, and the optimization of dietary and lifestyle modifications, show promise as new treatment strategies for improving metabolic abnormalities and alleviating the clinical symptoms associated with PCOS.

However, our current understanding of gut dysbiosis in the pathogenesis of PCOS remains relatively limited, and numerous research findings require further validation. Future studies should focus on elucidating the causal relationships and molecular mechanisms underlying the interactions between gut microbiota and PCOS, thereby providing more precise theoretical foundations for disease prevention and treatment.

Moreover, there is an urgent need to standardize and regulate techniques for detecting and intervening in gut microbiota, which will necessitate interdisciplinary collaboration among microbiology, metabolomics, bioinformatics, and other relevant fields. This collaboration is vital for establishing reliable technical platforms and comprehensive databases. Additionally, efforts should be directed toward enhancing the translational application of basic research outcomes in clinical settings, optimizing gut microbiota-based diagnostic and therapeutic strategies for PCOS, and developing safe and effective microbiotic preparations that can improve patient compliance and outcomes.

In conclusion, research on the role of the gut microbiome in PCOS is still in its early stages, and the transformation of our understanding and management of this condition has only just begun. Moving forward, as we gain a deeper understanding of the role of gut microbiota in PCOS and integrate multidisciplinary efforts to refine microbiological intervention strategies, we are likely to make significant strides in alleviating the suffering of PCOS patients and enhancing reproductive health and quality of life for women more broadly.
